# Charity or empowerment? The role of COVAX for low and middle‐income countries

**DOI:** 10.1111/dewb.12349

**Published:** 2022-03-20

**Authors:** Felicitas Holzer, Tania Manríquez Roa, Federico Germani, Nikola Biller‐Andorno, Florencia Luna

**Keywords:** capacity building, COVAX, global vaccine access, patent waivers, vaccine equity

## Abstract

What has the past reaction to the COVID‐19 pandemic taught us? We have seen that many low and middle‐income countries (LMICs) still lack access to vaccines, and it seems little progress has been made in the last few months and year. This article discusses whether the current strategies, most notably, vaccine donations by the international community and the COVID‐19 global access facility COVAX, offer meaningful solutions to tackle the problem. At the centre of our analysis, we compare the concepts of “donations” and “charity” with “vaccine equity” and the “empowerment” of poorer countries. We suggest that the achievement of fair global vaccine production requires that our global approach is supportive of the idea of empowerment. We, therefore, need structural reforms, which would most importantly include capacity building, to positively impact this goal and to take the interests of the global poor seriously.

## 1 INTRODUCTION

Even though vaccine scarcity in low and lower middle‐income countries has been a concern right from the beginning of the COVID‐19 pandemic, it seems little progress has been made in the last year.^1^ Almost 10 billion vaccine doses have been administered globally, over eight months after the first vaccine deliveries worldwide, and around 61% of the world's population has received at least one dose of a vaccine at this point. Breaking down the numbers, however, shows that only 10% of people living in low‐income countries (LICs) have received one dose of a vaccine,^2^ which means that health workers and vulnerable people in many poorer countries still lack immunization. The vast majority of the doses have so far gone to people in high and upper middle‐income countries.^3^ The current global vaccine allocation has created large health benefits in richer countries, while poorer countries had to bear the adverse consequences owing to low vaccine quantities. Most notably, there is a strong assumption among scientists that precisely the lack of sufficient vaccination coverage in many countries leads to new SARS‐CoV‐2 variants and increased and continuous outbreaks, which affects both rich and poor nations.^4^

**Figure 1:**
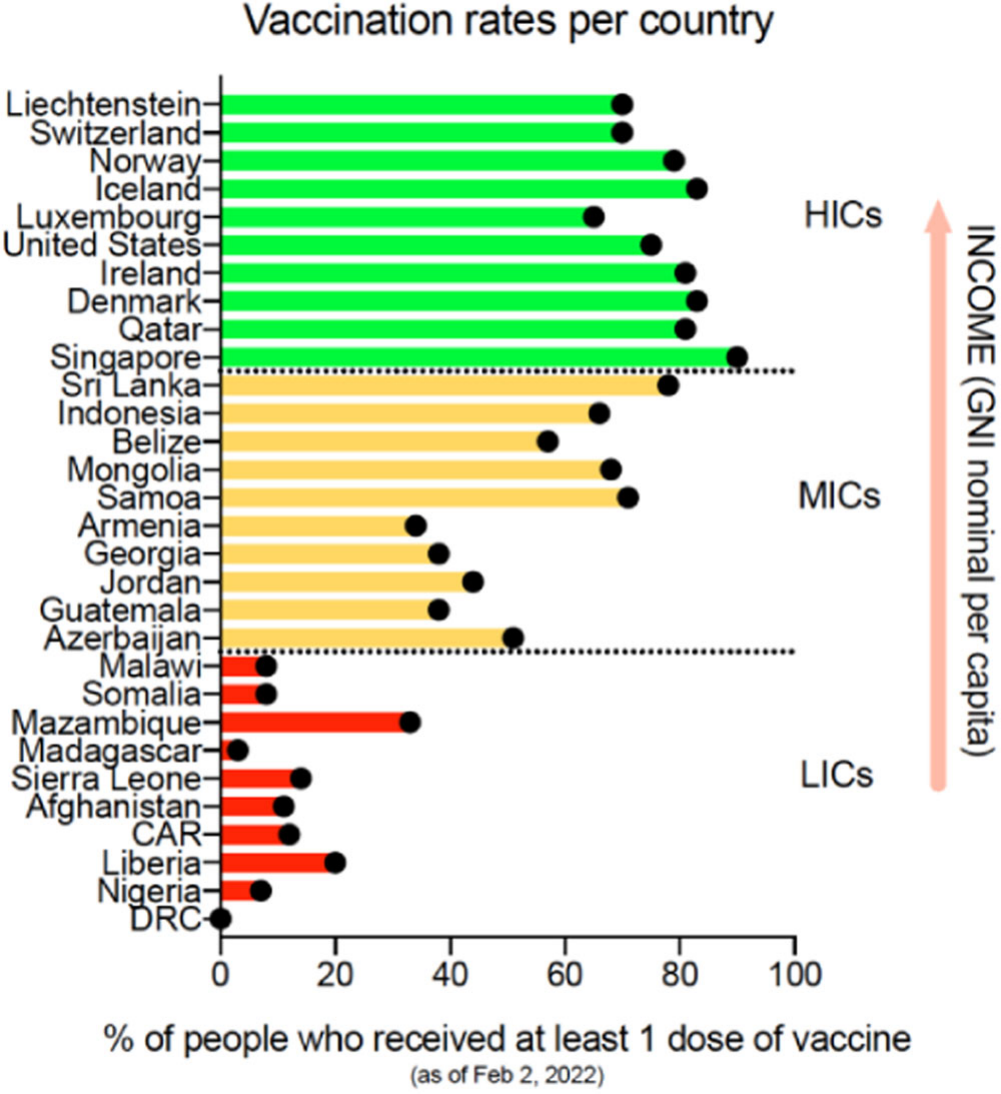
Percentage of people who received at least one COVID‐19 vaccine dose per country, in relation to their income (GNI nominal per capita). In green, the 10 HICs with the highest per capita income; in orange, the 10 MICs with the most average per capita income within thin category; in red, the 10 LICs with the lowest per capita income. Vaccination data are extracted from Our World in Data;^5^ Income (GNI nominal per capita) is obtained from publicly available World Bank data.^6^

In the following, we discuss whether the current strategies, most notably donations by the international community and the COVAX facility, offer meaningful solutions to solve the global vaccine production and access problem in low‐ and middle‐income countries (LMICs). At the centre of our analysis, we juxtapose the imperatives of “donation”, “charity” and “beneficence” on the one hand and “allocation”, “empowerment” and “equity” on the other. Eventually, we suggest that structural reforms, and most importantly capacity building, are required to positively impact the achievement of a fair global vaccine production and distribution that truly takes into consideration the interests of the global poor. In this context, we also provide a short analysis of the potential benefits related to patent waivers and their role as a catalyst to promote the empowerment of LMICs.

## 2 LESSONS LEARNED: BILATERAL DONATIONS AND THE COVAX FACILITY

Since many LMICs currently face a significant lack of vaccine access, G20 health ministers called in 2021 for more justice regarding the global vaccine rollout on their behalf.^7^ The G7 had previously agreed to donate 1 billion COVID‐19 doses to poorer countries in June 2021 and big pharma, including Pfizer and BioNTech, announced to produce 3 billion doses by the end of the year, intending to offer one third to the COVID‐19 global access facility COVAX.^8^ Following this rationale, the United States had committed to giving three‐quarters of its first tranche to the global access facility COVAX and European Union officials claimed to donate “many” of their surplus doses.^9^

However, there is still no consensus on vaccine donations to the developing world. Many nations have used bilateral donations to secure their geopolitical position and influence rather than advance global vaccine equity by sending the vaccines to those countries that need them most in terms of mortality or adverse economic effects of the COVID‐19 crisis. Some countries stand out in this regard: China, for instance, first and foremost made donations to allies that have participated in the Belt and Road Initiative; India fostered cooperation with countries to gain influence in the Asia‐Pacific region; and Russia gave vaccines to countries that were willing to purchase the Sputnik V vaccine.^10^ Vaccines have thus become, according to the author Vilasanjuan, a weapon in a geopolitical struggle.^11^ This has furthermore brought many poorer countries to a situation of dependency and disempowerment when it comes to accessing and manufacturing vaccines.

Donations have also been provided through the global cooperative scheme COVAX. The core idea of COVAX has been to set up a cooperative facility to accelerate the global vaccine development, production, and eventually the equitable access to COVID‐19 vaccines, tests, and treatments. Here, wealthier countries co‐finance those countries that only have limited access to vaccines and insufficient resources to enter into bilateral agreements with the manufacturers. As the first initiative ever to fight a pandemic by establishing a global vaccine allocation mechanism, it overcame the unfavourable history of vaccine development and allocation during last pandemics.^12^ In this sense, COVAX can be certainly considered a laudable enterprise.^13^ What distinguishes COVAX from the bilateral donations is most notably its aim, that is, the establishment of a fairness criterion; even if the current framework of a proportional fairness criterion is controversial.^14^ COVAX's distribution policy has been rightly criticized: a principle of equity has not been adequately taken into account (for example considering differential needs).^15^ Instead, COVAX' distribution criterion merely adhered to proportionality. It relies on a mechanism that ensures equal distribution proportionate to each country's population. It assumes that fair allocation requires treating differently situated countries identically, rather than equitably responding to each country's distinctive needs.^16^

COVAX as a mechanism has been an intermediate strategy for cooperation, where countries are still able to buy vaccines outside the facility and where international agreements to limit bilateral deals do not exist. Despite a strong December 2021, in which 300 million doses were shipped, the facility did not meet its own objectives to deliver 2 billion doses to low and middle‐income countries by the end of 2021.^17^ The current status quo shows that 1 billion COVID‐19 vaccines have been shipped to 144 participating countries.^18^

Despite its aim, serious difficulties hound COVAX. One problem is that the facility is chronically underfunded. To deliver on its full promise, the Access to COVID‐19 Tools (ACT) Accelerator,^19^ including COVAX, would need 16.8 billion USD.^20^ According to the latest release of the World Health Organization in March 2022, the ACT Accelerator has a funding gap of 15.7 billion USD regarding the 2021‐22 budget.^21^ Moreover, COVAX has been criticised for its insufficient coordination when it set up the facility, which led commentators to think that it failed to keep its promise to vaccinate the world.^22^

Cooperation also fails in the sense that many HICs with financial resources are still unproportionally prioritising their own populations and therefore hoarding vaccines that could be given to the COVAX facility and then administered to LMICs to effectively reduce the global burden caused by the pandemic.^23^ The view in favour of prioritising populations at the national level is commonly referred to as “vaccine nationalism”. This normative position sustains that governments ought to use law and other mechanisms to secure priority access to future vaccines. National governments have a primary duty to offer vaccines to their own citizens. There are different degrees of vaccine nationalism, reaching from rather extreme to moderate positions. The most extreme form of nationalism does not recognize any obligations toward people living outside the boundaries of nation states.^24^ This has sometimes been the implicit position of those HICs that have ordered and hoarded more vaccines than what their current populations needed through Advance Purchase Agreements.^25^

Given the great number of Advance Purchase Agreements and nationalistic tendencies placed under the motto “first come first served” on the global vaccine market, the participation of wealthier countries in a global distribution scheme remains a *pro forma* commitment at present. Already in late March 2021, the lack of funding for the COVAX facility seemed one of the central problems; and richer countries symbolically contributed to the facility to “soothe their conscience.”^26^ It seems little has changed ever since.^27^ That said, the prioritization of the domestic population from a country's perspective is to a certain extent understandable. In this regard, we tend to accept moderate vaccine nationalism as well as moderate cosmopolitanism.^28^ Our position recognizes special duties borne by governments towards its citizens and residents, and furthermore acknowledges general obligations to other persons in need.^29^ This, however, means to leave vaccine hoarding behind.

We can certainly learn from the COVID‐19 pandemic that the current vaccine production, procurement, and donation mechanisms alone achieve suboptimal outcomes at the global level. The pandemic has, for instance, revealed that countries in which manufacturers with patents are located, tend to hoard vaccines for their own populations, and poorer countries depend on their benevolence. Bilateral donations furthermore seem to be linked to geopolitical interests. This impairs the empowerment of especially those (mostly middle‐income) countries that could develop capacities to produce and adequately allocate vaccines themselves. If COVAX had followed its stated goals as it should have done, it could have offered an alternative beyond charity.

## 3 COVAX'S EXPANDED MANDATE: MOVING FROM CHARITY TO EQUITY AND EMPOWERMENT

As the philosopher and current adviser to the director‐general of the World Health Organization (WHO), Peter Singer, rightly points out, it is time to think about solutions beyond charity as an expression of the principle of benevolence.^30^ Charity still has an important role in our imperfect world, but it is not sufficient. Vaccine donations can only be one first step; the next one would be to systematically pursue vaccine equity, that is, to give LMICs a chance to fairly participate in the collective effort of vaccine manufacturing and procurement. Here, the underlying principle is the empowerment of LMICs.

To foster the empowerment of LMICs, it's worth looking at a pressing concern, that is, the slow rate of global *vaccine production* outside Europe and the US during the first two trimesters of vaccine rollout. Europe and the US together were responsible for most of the global vaccine exports. The diversification of production sites could be one of the key strategies to avoid extreme vaccine nationalism and bottleneck problems. One can assume that a higher number of production centres around the world would have a positive impact on the chances to get people vaccinated in LMICs, as vaccines would be less scarce. Obviously, this would not solve all coordination and infrastructure problems many LMICs still have, but could, nevertheless, be a very important first step. Apart from India, production facilities in LMICs barely exist up to the present day.^31^ In 2021, Africa has imported 99% of its vaccines while lacking the pre‐order purchasing capacity of richer nations.^32^

One of COVAX's important pillars is to scale up vaccine manufacturing abroad and thereby to strengthen capacities, but little has happened so far within the facility. In some middle‐income countries with strong research and development capacities, there is know‐how for manufacturing vaccines, which could be used more effectively. In turn, countries and regions with a basic or incipient research infrastructure may require support for technology transfer between innovators and targeted production sites. COVAX has so far been mainly a procurement coalition, but the facility should also have a stronger role in initiating and enabling a more effective and rapid technology transfer, as well as more systemic capacity building regarding both manufacturing and vaccine rollout.^33^

This may also benefit low‐income countries that do not yet possess the capacity to produce vaccines but may substantially benefit from the empowerment of middle‐income countries in their region. More specifically, a regional approach, where the wealthiest countries of a region take on production responsibilities for the very poor, might still be a step forward to achieve regional empowerment.

However, many pharmaceutical companies are currently hesitant to pursue this path. Citing reasons including quality concerns and the time required to get new companies up to speed are accompanied by efforts to ramp up their own production so that HICs and pharma can increase donations to the global poor. The main argument is that the know‐how and infrastructures needed to produce mRNA vaccines against COVID‐19 are not easily transferrable to other sites if this shall be done in a timely manner. Vaccine production must also meet the requirements for quality control, quality assurance, and regulatory oversight, which counts among other obstacles. Only few LMICs are currently in possession of the capacities necessary to produce these vaccines.^34^

Pfizer and BioNTech keep relying on the charity argument: the firms announced to provide 2 billion doses to countries until 2022, which means that pharma companies try to actively bolster supply to LMICs.^35^ But it seems that their initiative comes quite late, as many LMICs have lacked broader access to mRNA vaccines so far. Most people have gotten viral‐vector shots, that is, the AstraZeneca vaccine or inactivated vaccines, such as Sinovac.^36^ In addition, there has also been a problem regarding the timing of vaccine delivery, as countries would need to receive vaccines over a certain period and not all at once. In the latter case, health systems may get easily overburdened and vaccine administration would likely fail.^37^

In the same vein, Paul Stoffels, J&J's chief scientific officer, explained that transferring technology requires time and resources to train workers and to produce complex and new products in LMICs.^38^ This argument is, however, quite weak, given that capacity building could have started already in December 2020 if properly organised by pharma companies and the international community, which would have given the responsible authorities, pharma, and governments the necessary time to prepare vaccine manufacturing. Contrary to this trend, Russia, for instance, has been handing off the publicly financed and produced Sputnik V vaccine while giving instructions for the technology process. Russia broadly licensed the jab to 34 drug companies abroad, including India and Brazil. Several drug companies received essential ingredients from Russian scientists and lists of equipment and supplies. Russian scientists furthermore visited the plants to teach the workforce abroad the manufacturing process.^39^

This example proves that a systemic transfer of know‐how, ingredients, and capacity building can be a feasible enterprise despite the differences in the technology used. However, when accomplished unilaterally by single countries, there might be the general suspicion that dependencies can easily be created and maintained in the country receiving the new technology and capacity building. To seriously leave the rhetoric of beneficence behind, one would need to envision a cooperative initiative to truly empower countries that would acquire capacities regarding the manufacturing process independently from the geopolitical and financial interests of those countries in which the manufacturing facilities are located. We nevertheless acknowledge that vaccine technologies show different levels of complexity. In this regard, countries may not necessarily start by producing mRNA vaccines, which require a higher level of capacity building. But other vaccines have shown to produce very reliable results in protecting people from SARS‐CoV‐2 virus disease when it comes to administering them in LMICs.^40^ In what follows, we explore potential approaches to reform the current system.

## 4 EMPOWERING LMICS: THE ROLE OF PATENT WAIVERS

Facing the problem of pharma's reluctance towards the expansion of production to other sites, knowledge transfer, and systematic capacity building, US President Biden announced on May 5, 2021, his support to the international petition to waive intellectual property rights to vaccines for the duration of the COVID‐19 pandemic. His aim was to allow other countries to produce and thus globally speed up the production of Pfizer‐BioNTech and Moderna vaccines against COVID‐19 disease.^41^ This attempt has eventually been blocked by Japan, South Korea, the United Kingdom, and the European Union member states.^42^ Governments remained deeply divided over this effort, also months after Biden's announcement: While China, Russia, the United States, and the World Health Organization supported an IP waiver on vaccines, the pharmaceutical industry together with the countries blocking the initiative held the view that an IP waiver would not accelerate vaccine production.^43^ In this regard, many think that Biden's announcement has been a rather symbolic step.^44^ However, the European Union member states declared themselves open to negotiations despite Germany's strong opposition back then. A TRIPS (Trade‐Related Aspects of Intellectual Property Rights) waiver could be granted by a three‐quarters vote of WTO (World Trade Organization) members.

But as the “ALLEA Statement on Vaccination Bottlenecks in the Global South and a Patent Waiver for COVID‐19 Vaccines” from December 2021 shows, the common European view remains that patent waivers are not a silver bullet in the pursuit of vaccine equity. Instead, WTO members states actively pushing for patent waivers should rather consider improving procedures and institutional design or help streamline the process for compulsory licensing on pharmaceutical products.^45^ Meanwhile, EU had at least agreed on increased exports, a better use for compulsory licensing, and fewer export restrictions.^46^

The question one may ask is whether patent waivers by themselves solve the scarcity problem or whether they are (only) one element to help scale up production and empower LMICs by systematically strengthening their capacities.

According to Hotez et al., the drive for intellectual property waivers partly stems from past experiences with HIV/AIDS drugs, where patent pools, patent waivers, and other liberalizing mechanisms favourably supported the equitable access to lifesaving drugs.^47^ Yet, they point out that the debate about intellectual waivers of intellectual property rights still needs to be led with prudence. Even if waivers may have a positive effect on the global vaccine production, there is a potential caveat, as they may signal fewer incentives to innovators with respect to future pandemics. However, Hotez et al. also acknowledge that the proposal to timely limit waivers could mitigate this problem considerably.

Advocates put forward the argument that patent waivers (and their announcement) a priori increase the investments in LMICs and therefore manufacturing capacity to produce COVID‐19 vaccines while assuring that patents are not breached. This is in part backed up by the legal certainty that patent waivers create in the current multilateral trading system. Patent waivers also signal that governments in HICs are serious about increasing access to COVID‐19 vaccines.^48^ It is certainly true that the current number of countries that can enter in COVID‐19 vaccine production is limited, and capacity strengthening, especially in continents like Africa, Latin America, and Asia would be key to enhance production. This is, however, beyond the mere politics of patent wavers. But patent waivers can be a necessary step and may signal governments and institutions in LMICs to take the right measures towards the improvement of know‐how and infrastructure needed to produce vaccines. In this regard, they can positively support LMICs in their empowerment to overcome the current dependencies in the domain of global health.

Another key argument against patent waiver scepticism concerns the type of vaccine produced. Javier Guzman, technical director at Management Sciences for Health, put forward that the debate has mainly focused on mRNA vaccines, but more manufacturers in LMICs are in the position to produce viral vectors and/or to contribute with the fill‐and‐finish stage of the process in which sterile vials are filled and capped.^49^ In addition, conventional vaccines can increasingly be developed and produced, which could be another production source in middle‐income countries (MICs). Technologically advanced MICs, such as many Latin American countries, could further advance and get prepared to produce mRNA vaccines and begin to build the necessary infrastructure for future pandemics. For instance, Argentina has produced the active ingredient of the AstraZeneca vaccine and Mexico has overseen the fill‐and‐finish process.^50^ Now, these two countries and Brazil may also serve as a pilot when it comes to bolstering the mRNA vaccine production in LMICs.^51^

It would be also worth discussing different models of patent waivers and/or sub‐license agreements. For instance, pharma companies could give open licenses to other manufacturers (as it is currently done with the AstraZeneca vaccine) and be paid an initial amount of money for developing the drug in accordance with the obtained health impact.^52^

## 5 EMPOWERING LMICS: COVAX'S ROLE IN FACILITATING CAPACITY BUILDING AND TECHNOLOGY TRANSFER

Like the supporters of patent waivers, we think that temporarily suspended patents are a good first step to counteract dependencies of LMICs. Here, support should be provided especially to those LMICs that have an incipient infrastructure and manufacturing potential. While middle‐income countries are more likely to have the capacities to effectively start producing vaccines in the short‐term, low‐income countries without the necessary workforce and infrastructure would also benefit from neighbour countries that produce vaccines. The systematic strengthening of regional distribution mechanisms would be an important complementary measure to benefit especially low‐income countries. In this sense, patents should no longer be considered a “sacred cow” in times of a pandemic. Given that many (past and future) pandemics have concerned and will concern LMICs, this strategy would be crucial to accelerate the development of vaccines against emerging and existing infectious diseases. On the other hand, if patents shall still play a central role for the development of vaccines, COVAX's could take the lead in encouraging the development of patents in lower‐income countries; efforts that are currently not undertaken.^53^

Moreover, COVAX should, in our opinion, have a more active role in facilitating capacity building in LMICs and the technology transfer between vaccine innovators, i.e. pharmaceutical companies and potential manufacturers. CEPI (the Coalition for Epidemic Preparedness Innovations), one of the three institutions governing COVAX besides WHO and Gavi, is a crucial actor in assuring innovative global partnerships between public, private, philanthropic actors, and the civil society. CEPI continues accelerating the development of vaccines against emerging infectious diseases. But here, it must be made sure that equitable access to these vaccines is given during pandemics. Technology transfer, the access to reagents, and the existence and investment in production sites are of special importance.

We hold the view that patent waivers alone are not enough but only effective if implemented as a supporting strategy to global cooperative efforts, even if they are tied to time limits and public health emergencies. Multilateral approaches, such as COVAX, are therefore very important. We acknowledge that COVAX is one possible multilateral approach, but there could be alternatives. Still, COVAX under its current design is a good first step towards a multilateral mechanism, as the facility is committed to comply with principles of fairness when it comes to the global allocation of vaccines. Contrary to the aspiration of “mere charity” through bilateral donations that can create (intended or unintended) dependencies, COVAX adheres to a fairness criterion by offering proportional access to vaccines to all participating LMICs. The facility is certainly a good starting point that can progress fast and undergo further improvement. The WHO could take a lead role in cooperative efforts by improving and coordinating vaccine procurement, capacity building, and production, as well as by incentivizing countries to bring different stakeholders together. These lead institutions would furthermore be supported by institutions and processes with direct or indirect impact, including the UN and the World Trade Organization (WTO), and institutions created at national and regional levels to support and enable the global access to vaccines.^54^ Instead of supporting individual countries' interests through bilateral donations, COVAX could offer a chance to pursue a global strategy of empowerment, which would considerably go beyond its main current role as a procurement community dependent on the good‐will of HICs and their vaccine donations.

Following our previous line of argument, it is necessary to build and strengthen production capacities in different countries and regions, especially in those ones that possess a basic infrastructure to produce vaccines. A solution to empower LMICs is most likely to be found in the strengthening of international cooperation with the goal of working towards global equitable access to vaccines, facilitated by a multilateral structure, and with the support of other measures that help share knowledge and expertise, as needed.

## 6 CONCLUSION

In our paper, we outlined the problems associated with vaccine hoarding in HICs and international donations by the pharmaceutical industry and countries in possession of the production facilities. To tackle these shortcomings, we suggested that the focus of reform should be the improvement of global cooperation with the goal of ensuring equitable access to vaccines. We argue that regional capacity building for vaccine manufacturing, technology transfer, the support of patent waivers, and the improvement of local infrastructure will be crucial. Note that at the beginning of the vaccine distribution process, various problems arose in regions, such as Latin America and Africa, regarding the transportation, storage, and even the application of vaccines due to lacking infrastructure and know‐how. All these adversities were solved in a short time, which is an example of the learning process that these countries went through.^55^ Following this logic, we suggest that COVAX, or a similar multilateral agency, should be further strengthened and improved in its current mandate, which will subsequently empower LMICs in their manufacturing capacity. While in a globalised world it may be unrealistic and even unreasonable for every country to be self‐sufficient in producing vaccines, diversifying production sites regionally will nevertheless help the global community move away from a scheme based on the donations of rich countries to poorer countries.

## FUNDING

The study underlying this article is part of an Epidemic Ethics/WHO initiative which has been supported by FCDO/Wellcome Grant 214711/Z/18/Z. The authors alone are responsible for the views expressed in this publication and they do not necessarily represent the views, decisions or policies of the World Health Organization.

## Footnotes

1

World Bank . (2021). ‘Absolutely Unacceptable' COVID‐19 Vaccination Rates in Developing Countries. Retrieved January 12, 2022, from https://www.worldbank.org/en/news/podcast/2021/07/30/-absolutely-unacceptable-vaccination-rates-in-developing-countries-the-development-podcast.

2

Our World in Data . (2022). Coronavirus (COVID‐19) Vaccinations. Data retrieved January 20, 2022, from https://ourworldindata.org/covid-vaccinations

3
See Figure 1.

4

The Conversation . (2021). Are new COVID variants like Omicron linked to low vaccine coverage? Here's what the science says. Retrieved November 29, 2021, from https://theconversation.com/are-new-covid-variants-like-omicron-linked-to-low-vaccine-coverage-heres-what-the-science-says-170262.

5
Our World in Data, op. cit. note 2, Updated daily data retrieved January 30,2022, from https://ourworldindata.org/covid-vaccinations.

6

The World Bank . (2022). GNI per capita, Atlas method. Data retrieved January 30, 2022, from https://data.worldbank.org/indicator/ny.gnp.pcap.cd?year_high_desc=true

7

United Nations . (2021). WHO to G20 Health Ministers: Meet COVID‐19 pledges, support regional vaccine manufacturing. Retrieved September 6, 2021, from https://news.un.org/en/story/2021/09/1099212

8

Lee, J. , & Morton, B. (2021). G7: World leaders promise one billion COVID vaccine doses for poorer nations. BBC News. Retrieved September 20, 2021, from https://www.bbc.com/news/uk-57461640.

9

Guarascio, F. (2021). US will donate substantial portion of vaccines through COVAX – US official. USNews and World Report. Retrieved on September 20, 2021, from https://www.usnews.com/news/world/articles/2021-05-19/us-will-donate-substantial-portion-of-vaccines-through-covax-us-official; Bollyky, T. J., Murray, C. J., & Reiner, R. C. (2021). Epidemiology, not geopolitics, should guide COVID‐19 vaccine donations. The Lancet. 398(10295), 97‐99.10.1016/S0140-6736(21)01323-4PMC818684834115978

10
Ibid.

11

Vilasanjuan, R. (2021). COVID‐9: the geopolitics of the vaccine, a weapon for global security. Elcano Royal Institute. Retrieved September 10, 2021, from http://www.realinstitutoelcano.org/wps/portal/rielcano_en/contenido?WCM_GLOBAL_CONTEXT=/elcano/elcano_in/zonas_in/ari32-2021-vilasanjuan-covid-19-the-geopolitics-of-the-vaccine-a-weapon-for-global-security.

12
For instance, in 2009, the H1N1 virus killed almost 300,000 people even though a vaccine had been developed within seven months from the beginning of the pandemic. 90% of the total vaccine production was made accessible to ten high‐income countries. Only after negotiations with WHO, 10% of the vaccine doses were released to make them accessible to other countries. See Bollyky, T. J., & Bown, C. P. (2020). The tragedy of vaccine nationalism: only cooperation can end the pandemic. Foreign Affairs. 99, 96 Luna, F. , & Holzer, F. (2021). International cooperation in a non‐ideal world: the example of COVAX. Cadernos Ibero‐Americanos de Direito Sanitário, 10(3), 199–210.34938817

13

Manríquez Roa, T. , Holzer, F. , Luna, F. , & Biller‐Andorno, N. (2021). Expert Views on COVAX and Equitable Global Access to COVID‐19 Vaccines. International journal of public health, 66, 1–8.10.3389/ijph.2021.1604236PMC875855935035347

14

Emanuel, E. J. , Persad, G. , Kern, A. , Buchanan, A. , Fabre, C. , Halliday, D. , Heath, J. , Herzog, L. , Leland, R. J. , Lemango, E. T. , Luna, F. , McCoy, M. S. , Norheim, O. F. , Ottersen, T. , Schaefer, G. O. , Tan, K. C. , Wellman, C. H. , Wolff, J. , & Richardson, H. S. (2020). An ethical framework for global vaccine allocation. Science, 369(6509), 1309–1312.3288388410.1126/science.abe2803PMC8691258

15

Ibid, E. , et al. acknowledge that an equity criterion needs to be established for the fair distribution of vaccines. However, the authors have suggested another allocation framework, that is, the Fair Priority Model to advocate a needs‐based distribution instead of a proportional equity criterion. The needs‐based model considers three phases to reach a truly equitable distribution. First, it considers the reduction of premature deaths as a consequence of the health emergency; in a second phase, distribution is aimed at reducing economic hardship (with a focus on the overall economic improvement and the extent of people that would be spared from poverty); the third phase concerns the reduction of transmission rates to restore normalcy.

16
See Kok‐Chor Tan's remarks in “Philosophy Profs Discuss ethical COVID‐19 Distribution at Penn Science Café Event”. Retrieved February 25,2022, from https://www.thedp.com/article/global-vaccine-distribution-ethics-penn-science-cafe-event. We thank an anonymous reviewer for this point.

17

Akhtar, M. (2022). Is Covax finally going to vaccinate the world? Vox. Retrieved January 12, 2022, from https://www.vox.com/future-perfect/22872438/covax-omicron-covid-19-vaccine-global-inequity

18
GAVI: The Vaccine Alliance. (2022). COVAX has so far shipped over 1 billion COVID‐19 vaccines to 144 participants. Data retrieved January 20, 2022, from https://www.gavi.org/covax-vaccine-roll-out.

19
The ACT Accelerator is a global collaboration to accelerate the development, distribution and equitable access to COVID‐19 tests, treatments, and vaccines. The COVAX facility is part of the ACT Accelerator.

20

Focus2030 (2021). Financing the Global Response to COVID‐19: Which G20 Countries Play by the Rules? Retrieved August 20, 2021, from https://focus2030.org/Financing-the-global-response-to-Covid-19-which-G20-countries-play-by-the-rules; World Health Organization (2022). Access to COVID‐19 tools funding commitment tracker. Retrieved March 9, 2022, from https://www.who.int/publications/m/item/access-to-covid-19-tools-tracker.

21
Ibid: b.

22

Furneaux, R. , Goldhill, O. , & Davies, M. (2021). How COVAX failed on its promise to vaccinate the world. The Bureau of Investigative Journalism. Retrieved October 10, 2021, from https://www.thebureauinvestigates.com/stories/2021-10-08/how-covax-failed-on-its-promise-to-vaccinate-the-world.

23

Ibid, & Paremoer, L .(2021). Emanuel, E., Fabre, C., & Halliday, D., et al. (2021). How many vaccine doses can nations ethically hoard? Foreign Affairs. Retrieved October 20, 2021, from https://www.thecairoreview.com/essays/a-pandemic-of-vaccine-and-technology-hoarding-unmasking-global-inequality-and-hypocrisy/ https://www.foreignaffairs.com/articles/world/2021-03-09/how-many-vaccine-doses-can-nations-ethically-hoard

24

Emanuel, E. J. , Buchanan, A. , Chan, S. Y. , Fabre, C. , Halliday, D. , Leland, R. J. , Luna, F. , McCoy, M. S. , Norheim, O. F. , Schaefer, G. O. , Tan, K. C. , & Wellman, C. H. (2021). On the Ethics of Vaccine Nationalism: The Case for the Fair Priority for Residents Framework. Ethics and International Affairs. Ethics and international affairs, 35(4), 543–562.3493799010.1017/s0892679421000514PMC8691804

25

Holzer, F. , Luna, F. , Manriquez Roa, T. , & Biller‐Andorno, N. (2021). A matter of priority: equitable access to COVID‐19 vaccines. Swiss Medical Weekly, 151,w20488.3371514510.4414/smw.2021.20488

26
Manríquez Roa, et al., op.cit. note 13.

27
Paremoer, op. cit. note 23: b.

28
See Emanuel, et al., op. cit. note 24, p. 4. We agree with Emanuel et al.'s (2021) position that in their extreme forms, neither vaccine nationalism nor vaccine cosmopolitanism is ethically plausible. The first ignores the basic moral claims of human beings beyond a country's borders (it is wrong to ignore the suffering of others when it is in our power to help with little or no sacrifice or burden); the second overlooks a government's special obligations to their citizens.

29
For example the “fair priority for residents” framework or the flu risk standard; see Emanuel, et al., op. cit. note 24; Emanuel, et al., op. cit. note 14.

30

Maxmen, A. (2021). The fight to manufacture COVID vaccines in lower‐income countries. Nature, 597(7877), 455–457.3452669510.1038/d41586-021-02383-z

31

Guetta‐Jeanrenaud, L. , Poitiers, N. , & Veugelers, R. (2021). A world divided: global vaccine trade and production. Bruegel Blog Post. Retrieved October 10, 2021, from https://www.bruegel.org/2021/07/a-world-divided-global-vaccine-trade-and-production/.

32

Nature Editorial . (2021). A patent waiver on COVID vaccines is right and fair. Nature, 598,478.10.1038/d41586-021-01242-134035532

33
Manríquez Roa, et al., op. cit. note 13, p. 4.

34
Maxmen, op. cit. note 30 Karp, P. (2021). Pfizer warns Australia a COVID vaccine patent waiver could harm supply and safety. The Guardian. Retrieved January 20, 2022, from https://www.theguardian.com/australia-news/2021/may/13/pfizer-warns-australia-a-covid-vaccine-intellectual-property-patent-waiver-could-harm-supply-and-safety

35

Hopkins, J. S. (2021). Pfizer, BioNTech to Deliver 2 Billion Covid‐19 Vaccine Doses to Developing Countries.The Wall Street Journal. Retrieved October 10, 2021, from https://www.wsj.com/livecoverage/covid-2021-05-21/card/GsPYoFscRppTzYYt0l4f.

36
Maxmen, op. cit. note 30.

37
We acknowledge that manufacturing processes may significantly differ depending on the vaccine type produced; see Guetta‐Jeanrenaud, et al., op. cit. note 31.

38
Maxmen, op.cit. note 30.

39
We acknowledge at this point that the manufacturing of Sputnik V might be easier compared to other vaccines; see Maxmen, op. cit. note 30.

40

Choi, E. M. (2021). COVID‐19 vaccines for low‐and middle‐income countries. Transactions of the Royal Society of Tropical Medicine and Hygiene, 115(5), 447–456.3373366310.1093/trstmh/trab045PMC7989148

41

Hotez, P. J. , Bottazzi, M. E. , & Yadav, P. (2021). Producing a Vaccine Requires More than a Patent. Intellectual Property is Just One Piece of an Elaborate Process. Foreign Affairs. Retrieved January 20, 2022, from https://www.foreignaffairs.com/articles/united-states/2021-05-10/producing-vaccine-requires-more-patent.

42
Nature Editorial, op. cit. note 32.

43

International Institute for Sustainable Development (IISD) (2021). WTO Chief Hopeful for Deal to Get More COVID‐19 Jabs to Developing Nations. Retrieved October 10, 2021, from https://sdg.iisd.org/commentary/policy-briefs/wto-chief-hopeful-for-deal-to-get-more-covid-19-jabs-to-developing-nations/.

44

Crützen, C. J. , & Kücking, M. (2021). The Waiver of Patent Protection for COVID‐19 Vaccines — On Practicability and Purpose of Such Measure. Mayer | Brown. Retrieved January 20, 2022, from https://www.mayerbrown.com/en/perspectives-events/publications/2021/07/ger-the-waiver-of-patent-protection.

45

World Trade Organization (2021). ALLEA Statement on Vaccination Bottlenecks in the Global South and a Patent Waiver for COVID‐19 Vaccines. Retrieved January 20, 2022, from https://www.wto.org/english/tratop_e/covid19_e/allea_letter_e.pdf

46

Rubin, H. , & Saidel, N. (2021). Innovation beyond patent waivers: Achieving global vaccination goals through public‐private partnerships. Brookings. Retrieved October 10, 2021, from https://www.mayerbrown.com/en/perspectives-events/publications/2021/07/ger-the-waiver-of-patent-protection.

47
Hotez, et al., op. cit. note 41.

48

Iacobucci, G. (2021). COVID‐19: How will a waiver on vaccine patents affect global supply? BMJ. 373, n1182; Zarocostas, J. (2021). What next for a COVID‐19 intellectual property waiver? The Lancet, 397(10288), 1871–1872.10.1136/bmj.n118233972288

49
Ibid.

50

Loewy, M. A. (2020). Argentina y México se unen para fabricar una vacuna “a riesgo” contra COVID‐19. Medscape. Retrieved August 13, 2020, from https://espanol.medscape.com/verarticulo/5905794#:%7E:text=Compa%C3%B1%C3%ADas%20farmac%C3%A9uticas%20de%20Argentina%20y,con%20la%20financiaci%C3%B3n%20del%20proyecto

51

Pan American Health Organization (PAHO) (2021). La OPS selecciona centros en Argentina y Brasil para desarrollar vacunas de ARNm contra la COVID‐19. Retrieved January 10, 2022, from https://www.paho.org/es/noticias/21‐9-2021-ops-selecciona-centros-argentina-brasil-para-desarrollar-vacunas-arnm-contra#:%7E:text=Washington%20DC%2C%2021%20de%20septiembre,y%20a%20futuro.

52

See Banerjee, A. , Hollis, A. , & Pogge, T. (2010). The Health Impact Fund: incentives for improving access to medicines. The Lancet, 375(9709), 166–169.10.1016/S0140-6736(09)61296-420109894

53
We would like to thank the anonymous reviewer for their comment.

54

Frenk, J. , & Moon, S. (2013). Governance challenges in global health. The New England Journal of Medicine, 368(10), 936–942; Van de Pas, R., Hill, P. S., Hammonds, R., Ooms, G., Forman, L., Waris, A., et al. (2017). Global health governance in the sustainable development goals: Is it grounded in the right to health? Global Challenges. 1(1), 47‐60.2861625510.1002/gch2.1022PMC5445596

55
We want to thank the anonymous reviewer for their comment.

